# A pooled analysis of risk factors of surgically treated leiomyosarcoma of the colon in adults

**DOI:** 10.1186/s12957-020-01838-3

**Published:** 2020-03-28

**Authors:** Yun Wang, Hao Wang, Zhi-Lu Yuan, Jing-Fei Zhao, Dian-Bo Dong, Qian Gao

**Affiliations:** 1 Department of Digestive System, Qinghai University Affiliated Hospital, Xining, 810000 Qinghai China; 2 Qinghai University, Xining, 810000 Qinghai China; 3 Department of Intensive Care Medicine, Qinghai Province People’s Hospital, Xining, 810007 China; 4 Department of General Surgery, Liao Cheng The Third People’s Hospital, Liaocheng, 252000 Shandong People’s Republic of China; 5 Department of Anorectal Medicine, Liao Cheng People’s Hospital, Liaocheng, 252000 Shandong People’s Republic of China

**Keywords:** Leiomyosarcoma of the colon, Prognostic factors, Treatment

## Abstract

**Background:**

This current systematic review aimed to evaluate the role of surgical management and risk factors by pooled cases from all identified patients with colonic leiomyosarcomas.

**Methods:**

The authors searched the Ovid MEDLINE, Embase, PubMed, and Cochrane databases using the keywords “colonic,” “colon,” and “leiomyosarcoma.” Risk factors of colonic leiomyosarcoma in the pooled cohort were also evaluated.

**Results:**

Between 1923 and 2019, 41 cases of colonic leiomyosarcoma were identified in 22 (53.7%) males and 19 (46.3%) females, with a mean and median age of 58.7 ± 2.2 years and 56.0 years. According to univariate analysis, smaller tumor size < 8 cm was significantly associated with longer progression-free survival (HR = 6.957, 95% CI 1.405–34.442; *p* = 0.017), and younger age < 60 years was trending toward better overall survival (HR = 2.765, 95% CI 0.924–8.272; *p* = 0.069).

**Conclusions:**

Colonic leiomyosarcomas are rare neoplasms with aggressive clinical behaviors. Age < 60 years and tumor size < 8 cm were favorable factors for patients’ better survival.

## Introduction

In 1923, Scott firstly reported one case of colonic leiomyosarcoma (CLMS) [1]. Gastrointestinal LMS of the colon, an uncommon condition that accounts for less than 1% of all colorectal malignancies, has a strong propensity to recur and to metastasize at distant sites (liver and lung) [2]. Surgery and adjuvant chemotherapy have been used in the treatment of CLMS patients. But given the rarity of this tumor, there was no clear information about the risk factors following therapeutic strategy. Therefore, we performed an extensive literature review, and this current systematic review aimed to evaluate the role of surgical management and risk factors by pooled cases from all identified patients with CLMS [2–29].

## Methods

### Literature search strategy

**Fig. 1:**
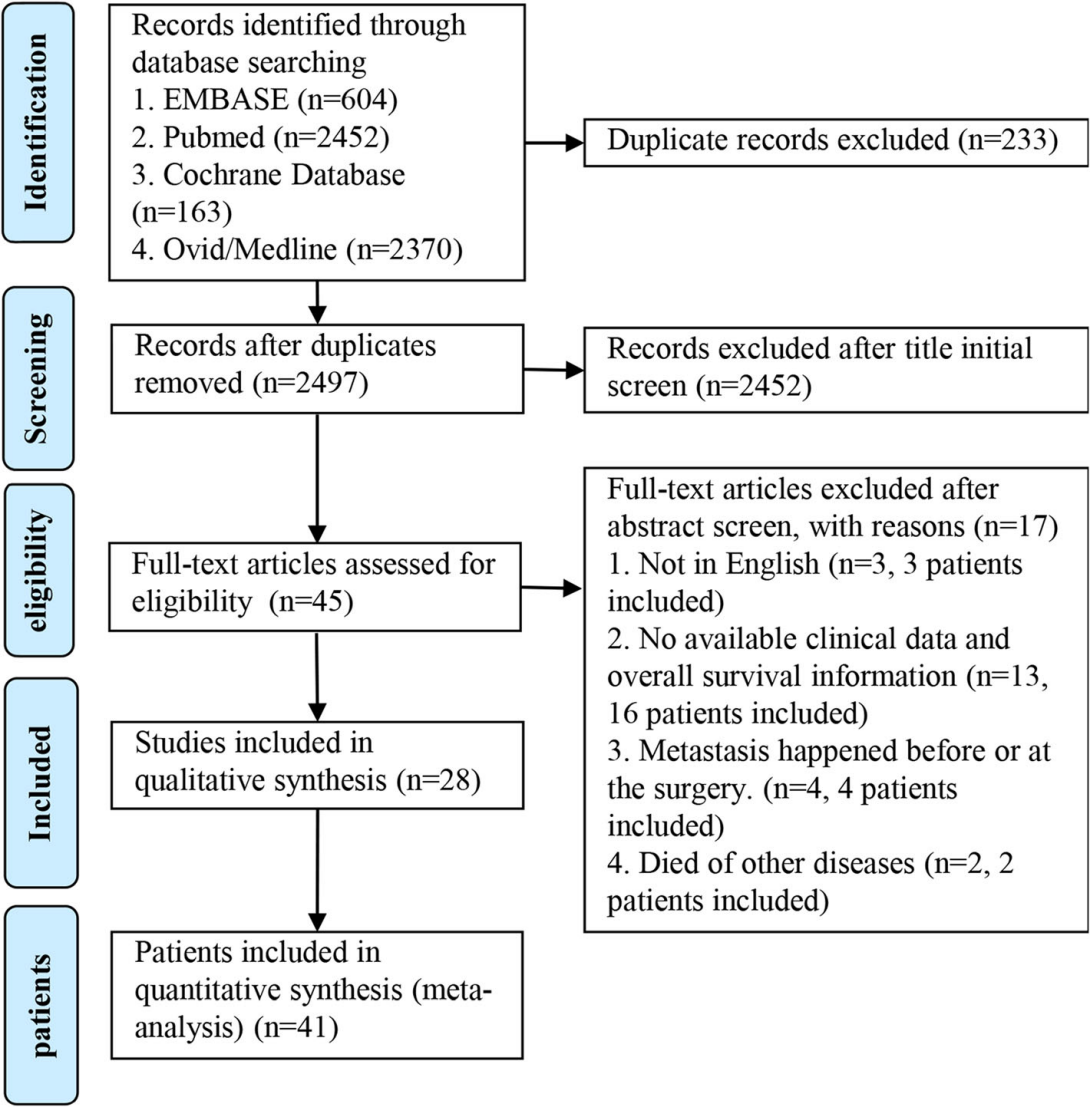
PRISMA flow diagram showing the inclusion and exclusion process for the analysis

The search protocol, including the search questions and inclusion and exclusion criteria, was developed a priority according to the Preferred Reporting Items for systematic reviews and meta-analysis (PRISMA) guidelines. We performed a systematic literature research of the Ovid Medline, Embase, Pubmed, and Cochrane Library Database from 1923 to 2019. The keywords used in the search were “colonic,” “colon,” and “leiomyosarcoma.” We further reviewed all the references provided in the publication and incorporate the pertinent citations. Language was limited to English. The process was shown in Fig. 1.

### Case eligibility criteria

Inclusion criteria for literature cases were as follows: (1) adult patients (≥ 18 years of age) with a diagnosis of CLMS who underwent surgery; (2) availability of overall survival (OS) data. Exclusion criteria for literature cases were as follows: (1) studies published in a language other than English; (2) unavailable patient data; (3) basic research rather than clinical report without clinical data; (4) patients have developed a distant metastasis before or at surgery; (5) patients died of other causes.

### Study eligibility and data extraction

Two investigators independently and in duplicate performed title and abstract screening of the studies in the search query results. Discrepancies between the review authors over the bias in studies were resolved by discussing with a third reviewer (Q.G) when needed. The following data were extracted from each study whenever possible: author and year of published articles, patient characteristics (gender and age), tumor size, treatment strategy, primary tumor gross appearance, duration of follow-up, progression-free survival (PFS), and OS. Due to incomplete and limited patients’ status for recurrence and metastasis, we defined PFS for both local and distant recurrence.

### Statistical analysis

Mean values are presented with their standard deviation (SD). The primary outcome of CLMS was PFS and OS, and its pertinent adverse factors were evaluated by univariate analysis. Due to the small number of patients, we did not perform multivariate Cox regression analysis. Outcomes were PFS and OS in subgroups with significant risk factors and their pertinent estimated PFS and OS time performed using the Kaplan-Meier method. All analyses were performed using IBM SPSS (version 25.0, IBM Corp.) with significance set at *p* < 0.05.

## Results

### Data from literature cases

From the period between 1923 and 2019, 41 cases of CLMS were identified in 22 (53.7%) males and 19 (46.3%) females, with a mean and median age of 58.7 ± 2.2 years and 56.0 years; respectively. The most common preoperative symptoms were abdominal pain (*n* = 15, 36.6%), followed by abdominal mass (*n* = 5, 12.2%), rectal bleeding (*n* = 3, 7.3%), bloody stool (*n* = 3, 7.3%), occlusion (*n* = 2, 4.9%), anemia (*n* = 2, 4.9%), lower gastrointestinal bleeding (*n* = 1, 2.4%), drowsiness (*n* = 1, 2.4%), alternating constipation (*n* = 1, 2.4%), and asymptomatic (*n* = 1, 2.4%). Tumor size was identified in 31 patients, ranging from 1.0 to 30.0 cm, with a mean and median size of 9.1 ± 1.2 cm and 7.8 cm, respectively. The gross appearance was identified in 23 patients. The most frequent appearance was polypoid (*n* = 8, 34.8%), followed by intramural (*n* = 5, 21.7%), type 2 (*n* = 4, 17.3%), exophytic (*n* = 2, 8.7%), pedunculate (*n* = 1, 4.3%), plaque (*n* = 1, 4.3%), sessile (*n* = 1, 4.3%), and subserosal (*n* = 1, 4.3%). Five patients in prior studies underwent open partial colectomy. No patients received radiotherapy prior to or after surgery. Only 2 (4.9%) patients underwent adjuvant chemotherapy after surgery (Table 1).

**Table 1:** Clinical characteristic of 41 cases with leiomyosarcoma of the colon

Authors and year	Sex/age (years)	Location	Symptom	Diameter (cm)	Treatment	Gross appearance	Recurrence/metastasis	Survival (months)
Golden T and Stout AP. [1]	M/78	S	Abdominal pain	NA	Surgery	NA	−/−	Alive, 12
MacKenzie DA et al. [4]	M/72	A	Abdominal pain	NA	Surgery	NA	−/−	Alive, 21
MacKenzie DA et al. [4]	F/57	T	Abdominal pain	NA	Surgery	NA	−/−	Alive, 21
MacKenzie DA et al. [4]	F/56	T	Abdominal pain	NA	Surgery	NA	−/−	Alive, 108
MacKenzie DA et al. [4]	F/55	S	Abdominal pain	NA	Surgery	NA	NA/NA	Dead, 6
Marshall SF and Cherry JW [5]	F/43	T	Abdominal mass	21	Surgery	NA	NA/NA	Dead, 10
Marshall SF and Cherry JW [5]	F/60	S	Abdominal mass	7.5	Surgery	NA	NA/NA	Dead, 14
Rogers V [6]	M/39	A	Abdominal pain	3	Surgery	Polypoid	−/−	Alive, 14
Lookanoff VA and Tsapralis PC [7]	M/77	A	Drowsiness	NA	Surgery	NA	NA/NA	Dead, 5
Yamakawa T and Hasebe M [8]	F/46	T	Abdominal mass	8.5	Surgery	NA	−/−	Alive, 20
Monga NK et al. [9]	F/45	A	NA	30	Surgery	NA	−/−	Alive, 6
Astarjian NK et al. [10]	F/74	A	Bloody stools	10	Surgery	NA	+/−	Alive, 16
Rao B K et al. [11]	F/58	A	Abdominal pain	17	Surgery	NA	−/multiple regions	Alive, 26
Stavorovsky M et al. [2]	F/52	T	Abdominal pain	12	Surgery	NA	−/−	Alive, 60
Suzuki A et al. [12]	M/33	D	Bloody stool	13.5	Surgery	Dumbbell	+/−	Dead, 8
Iwasa K et al. [13]	F/70	A	Abdominal pain	10	Surgery	Intramural	NA/NA	Alive, 12
Luna-Pérez P et al. [14]	F/44	C	Abdominal pain	NA	Surgery	NA	+/peritoneum, liver	Dead, 18
Luna-Pérez P et al. [14]	M/47	T	Occlusion	NA	Surgery	NA	−/−	Alive, 6
Miettinen M et al. [15]	M/54	D	Rectal bleeding	3.2	Surgery	Polypoid	NA/NA	Dead, 37
Miettinen M et al. [15]	M/61	A	Rectal bleeding	4.2	Surgery	Sessile	−/−	Alive, 141
Miettinen M et al. [15]	M/75	A	Anemia	6.5	Surgery	Plaque	NA/NA	Dead, 6
Miettinen M et al. [15]	F/76	C	Anemia	7.8	Surgery	Multinodular	NA/NA	Dead, 7
Miettinen M et al. [15]	F/36	S	Alternating constipation	6.5	Surgery	Polypoid	−/lung	Dead, 38
Miettinen M et al. [15]	M/66	A	Abdominal mass	NA	Surgery	Polypoid	−/liver	Dead, 19
Miettinen M et al. [15]	M/41	C	Rectal bleeding	7.5	Surgery	Pedunculated	−/humerus	Alive, 185
Miettinen M et al. [15]	M/65	D	NA	10	Surgery	Polypoid	−/visceral	Dead, 28
Michalopoulos A et al. [15]	F/67	T	Occlusion	5.7	Surgery	Polypoid	−/−	Alive, 12
Resch T et al. [18]	M/56	C	NA	NA	Surgery	NA	−/liver	Alive, 68
Yamamoto H et al. [19]	F/94	D	NA	25	Surgery	Type 2	−/liver	Dead, 7
Yamamoto H et al. [19]	M/56	S	NA	1	Surgery	Intramural	−/−	Alive, 60
Yamamoto H et al. [19]	F/78	S	NA	8.5	Surgery	Type 2	−/lung	Dead, 16
Yaren A et al. [24]	F/66	T	Abdominal pain	4	Surgery + chemo	Polypoid	−/−	Alive, 33
Samie A et al. [24]	M/65	S	Abdominal pain	2.7	Surgery	NA	−/−	Alive, 12
Kono M et al. [22]	M/46	T	Abdominal pain	11.8	Surgery	Type 2	+/liver	Alive, 30
Kiran P et al. [23]	M/54	A	Abdominal pain	13	Surgery + chemo	Intramural	+/−	Alive, 6
Janevski V et al. [24]	M/59	A	NA	10	Surgery	Exophytic	−/−	Alive, 8
Kim VM et al. [25]	M/82	C	Painless melenic stools	2.2	Surgery	Polypoid	−/−	Alive, 14
Akutsu D et al. [26]	F/51	D	Abdominal mass	4	Surgery	Type 2	−/−	Alive, 31
Yang J et al. [27]	F/55	A	Abdominal pain	8	Surgery	Exophytic	−/−	Alive, 5
Devriendt S et al. [28]	M/53	S	Asymptomatic	3.8	Surgery	NA	−/−	Alive, 15
Yahagi M et al. [29]	M/46	S	Hematochezia	4.2	Surgery	Polypoid	−/−	Alive, 17

*A* ascending colon, *C* cecum, *D* descending colon, *F* female, *M* male, *T* transverse colon, *S* sigmoid colon, *NA* not available

### Survival

**Fig. 2:**
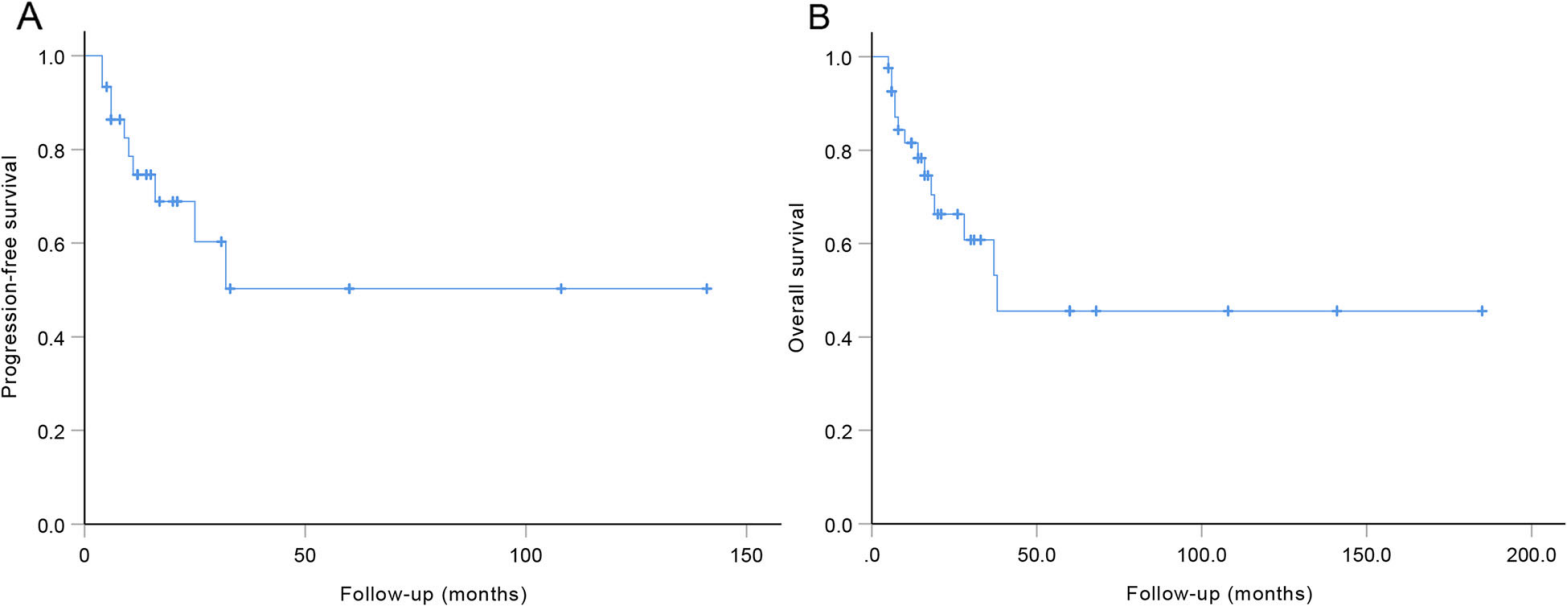
**a** PFS rates. **b** OS rates

In prior studies, patients’ status (*n* = 11) about recurrence and metastasis were not available. Four patients developed recurrence, and 7 patients happened to distant metastasis. The PFS rates of 30 patients with CLMS at 1, 3, and 5 years from the time of diagnosis were 74.6%, 50.2%, and 50.2%, respectively (Fig. 2a). The mean PFS was 79.0 ± 15.9 months. The OS rates of the entire series of patients with CLMS (*n* = 41) at 1, 3, and 5 years from the time of the diagnosis were 81.6%, 60.8%, and 45.6%, respectively (Fig. 2b), and the mean OS was 95.5 ± 18.6 months (Table 2).

**Table 2:** Clinical data and survival in literature cases

Variable	*n* (%)	Progression-free survival* (%)		*p* value	Overall survival (%)		*p* value
		1 year	3 year		1 year	3 year	
Gender				0.554^†^			0.252^†^
Male	22 (53.7)	68.6	45.8		85.9	66.2	
Female	19 (46.3)	83.1	54.5		76.7	54.9	
Age, yrs				0.560^†^			0.129^†^
≥ 60	17 (41.5)	80.0	60.0		76.5	31.1	
< 60	24 (58.5)	71.5	44.7		85.3	78.7	
Tumor size (cm)				**0.045**^†‡^			0.602^†§^
Range	1–30						
≥ 8.0	15 (51.7)	49.4	18.5		76.2	33.3	
< 8.0	14 (48.3)	91.7	68.8		80.0	72.0	
Tumor location				–			–
Ascending	13 (31.7)	87.5	32.8		83.9	62.9	
Transverse	9 (22.0)	85.7	85.7		87.5	87.5	
Descending	5 (12.2)	33.3	33.3		60.0	40.0	
Sigmoid	9 (22.0)	85.7	42.9		88.9	55.6	
Cecum	5 (12.2)	33.3	33.3		80.0	53.3	
Reports period				0.350^†^			0.594^†^
1923–1999	18 (43.9)	91.7	52.4		76.2	58.8	
2000–2019	23 (56.1)	63.6	47.7		85.9	64.9	

Bold means *p* < 0.05

*Eleven patients were excluded

^†^Chi-square test

^‡^Seventeen patients were excluded

^§^Ten patients were excluded

**Fig. 3:**
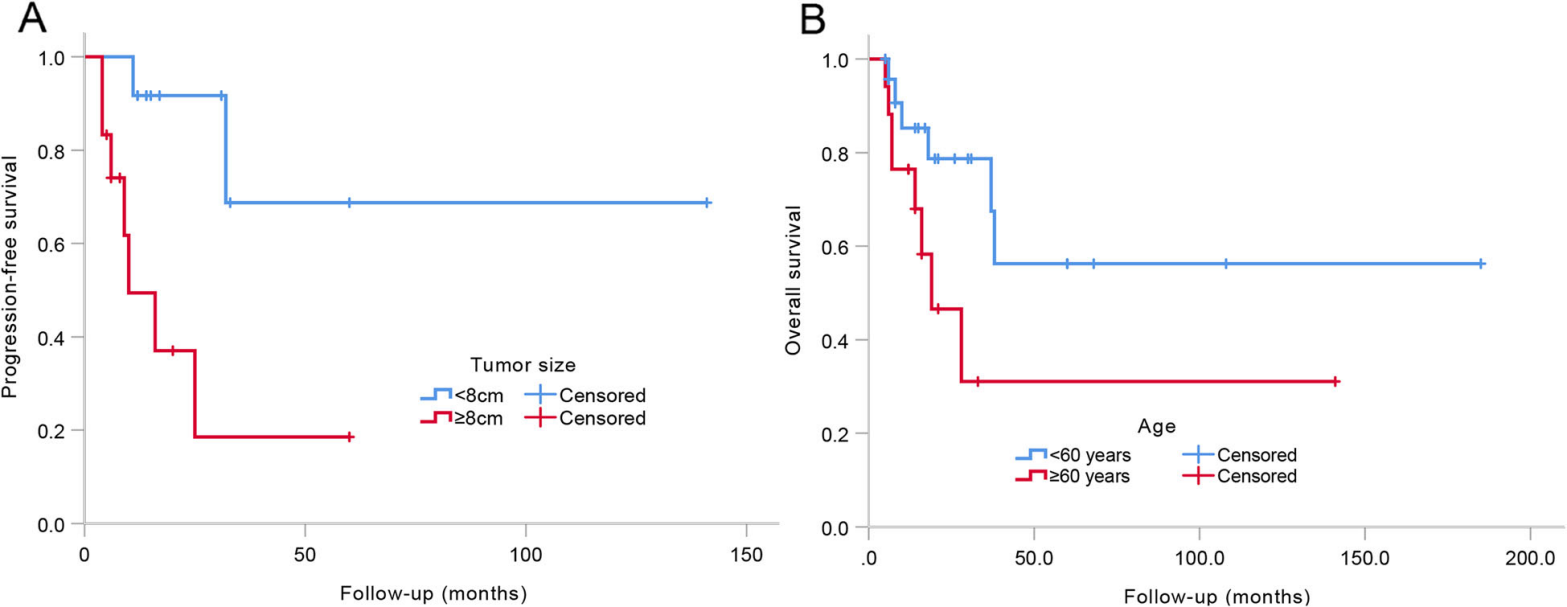
**a** Different PFS between tumor size < 8 cm and tumor size ≥ 8 cm. **b** Different OS between age < 60 years and age ≥ 60 years

According to univariate analysis, smaller tumor size < 8 cm was significantly associated with improved PFS (HR = 6.957, 95% CI 1.405–34.442; *p* = 0.017) (Fig. 3a), and younger age < 60 years was associated with better OS (HR = 2.765, 95% CI 0.924–8.272; *p* = 0.069) (Fig. 3b) (Table 2).

## Discussion

CLMSs are extremely rare neoplasms, and most of them have been described as case reports. In the past, LMS of the colon’s prognosis has been generally considered to be a benign tumor that displayed optimism with a low propensity for recurrence and distant metastasis [1, 30]. Later on, literature reported that frequent recurrences and distant metastasis have been observed in the CLMS [31]. Due to the paucity of data about CLMS, the information regarding its clinical characteristics and specific treatment was still unclear. Based on prior studies, we identified influencing risk factors for PFS and OS after surgical treatment, and aimed to increase the better understanding of this type of tumor.

LMS of the colon is slightly more frequent in males. Rao BK et al. reviewed 42 cases with CLMS that female dominance was found in his study [11]. The mean age in this study at the time of diagnosis was 56 years old, which is older than that in a literature review that reported a mean age at diagnosis of 50 years old [13]. Meanwhile, we found that older people had a decreased OS.

Based on the only complains and physical examinations, it is difficult to make an identified diagnosis of CLMS because preoperative symptoms, such as pain, diarrhea, and constipation, are insufficient evidence to make a diagnose of CLMS [12]. LMS could be exactly confirmed by the expression of smooth muscle actin and lack of CD117 [32].

Warkel et al. reported that survival of patients with the CLMS was not associated with the tumor size, but with mitotic activity [31]. In contrast, our study indicated that larger tumor size was associated with worsened PFS. One previous study consistent with our study advocated significant association between large tumor size and poor survival [33]. Unfortunately, with unavailable and incomplete data regarding mitotic activity, we failed to identify the relationship between mitotic activity and survival.

Surgery had been generally considered as the first line treatment for patients with CLMS. EH Ng et al. [34] published a review of 191 patients treated with surgery, and those who underwent complete resection has 25 months longer OS (the median time) than those with incomplete. In our series, due to the lack of details about the type of resection in the operative report of resection, we were unable to find significant difference in PFS or OS rates among patients who received different surgical treatments.

One finding of concerns in this study was the extremely high metastasis rate, and the most frequent sites of distant locations mainly in the liver, lung, peritoneum, humerus, and viscera. Even, many reviewers reported that CLMS with an aggressive clinical behavior that tends to high recurrence and metastasis after radical surgery [33, 35]. Seven patients developed distant metastasis in this current review study, in addition, many patients have developed metastasis before they underwent surgery. Patients with a distant metastasis in general had a poor survival. Therefore, adjuvant treatment after surgery might be recommended in patients with malignant tumors. To the Best of Our Knowledge, however, no postoperative radiotherapy for LMS of the colon has been reported yet. Adjuvant chemotherapy has been described in only two studies, with mixed results. Yaren A et al. [20] reported that a 66-year-old female with CLMS underwent adjuvant chemotherapy with ifosfamide plus doxorubicin after surgery, and no evidence of disease was observed during his follow-up time. Kiran P et al. [23] reported that a 54-year old male with LMS of the colon received postoperative chemotherapy with ifosfamide and doxorubicin for six cycles, but then he developed a recurrence after a disease-free period of half a year. After a surgery for recurrence, he was still alive well without disease. With the two better results described, adjuvant chemotherapy following surgery might be optimal for patients with large LMS of the colon. However, we could not definitely confirm the role of it because of inadequate follow-up time and limited cases. Longer follow-up could be performed to identify the effect of adjuvant treatment.

## Limitations

The limitations of this study were as follows: (1) the major was its retrospective nature, and selection bias always played a role; (2) the assessment methods of surgical management were undetailed described among studies, and we were unable to define which type of surgery did favor to increased survival; 3) we did not give an identified answer to a question whether better survival was beneficial from adjuvant therapy.

## Conclusions

CLMS are rare neoplasms with aggressive clinical behaviors, with a mean OS of 95.5 ± 18.6 months. Some potential risk factors were associated with worse survival; younger age ≥ 60 years and tumor size ≥ 8 cm were associated with patients’ decreased survival. Surgery followed by chemotherapy is recommended as the optimal treatment for CLMS. Given the rarity of this tumor, a prospective multiple-center randomized control trial should be performed.

## Data Availability

Not applicable.

## Abbreviations

CLMS: Colonic leiomyosarcoma
LMS: Leiomyosarcoma
OS: Overall survival
PFS: Progression-free survival
SD: Standard deviation
